# Prevalence of Postpartum Depression among Mothers in the Emirates of Abu Dhabi

**DOI:** 10.1007/s10995-024-03931-5

**Published:** 2024-06-29

**Authors:** Salma Al Ahbabi, Ghada Mubarak, Sharifa Al Khaldi, Ali Bin Mousa, Latifa Mohammad Baynouna Al Ketbi

**Affiliations:** Abu Dhabi Healthcare Services, Ambulatory Healthcare Services, Al Ain, United Arab Emirates

**Keywords:** Postpartum Depression, Edinburgh Postpartum Depression Scale EPDS, Prevalence, Determinants

## Abstract

**Background:**

Postpartum depression (PPD) is a common mental health condition that affects women in a silent and covert way and is not clearly visible to the community or to health care providers. Untreated PPD has significant and long-term consequences on the mother and their child. This study aims to assess the risk of postpartum depression among women in the Emirate of Abu Dhabi and its determinants.

**Method:**

This is a questionnaire-based cross-sectional study conducted at primary healthcare centers in the Emirate of Abu Dhabi. The target population is women visiting the well-child vaccination clinics for their infants’ vaccination. The questionnaire used consisted of socio-demographic characteristics, important histories such as obstetric, medical, and social histories, and the Edinburgh Post Partum depression scale EPPS. EPPS is a validated tool used to evaluate the probability of postpartum depression.

**Results:**

The probability of postpartum depression for women visiting the well child care clinics in the Emirate of Abu Dhabi during the study period was 35%, One-third. 10% had high risk, 7% had moderate risk, and 18% had mild risk. Using logistic and linear regression, there was an association identified between postpartum depression risk and the presence of weight concern and employment status OR 5.499(2.618–11.548) and OR 0.483 (0.246–0.951), respectively (*P* < 0.005). From the total sample, 3.7% responded quite often or sometimes to the question of having the intention to harm themselves.

**Conclusion:**

EPDS is recommended to be used routinely to screen women in the postnatal period. This high prevalence of risk of postpartum depression in the UAE (One in three women) calls for a well-prepared healthcare system and community. Healthcare providers need to be prepared with better knowledge, practice, and management strategies to care for these women, for early identification and management. Further studies should be undertaken to achieve effective strategies to reduce the incidence of this condition.

## Significance

Successful strategies for the prevention of postpostpartum depression and its early prevention and management are dependent on good research studies. This study revealed that the prevalence is almost one-third among women attending the well-baby clinics in Abu Dhabi with important associations identified. These findings call for action research aiming to prevent the condition or mitigate its impact on women, families, and the community.

## Introduction

Screening pregnant and postpartum women for depression reduces the risk of depression with or without additional treatment components compared with usual care (O’Connor et al., 2016). With the increasing prevalence of depression, including among pregnant and postnatal populations the impact on healthcare outcomes in postpartum women is valuable. According to Mathers and Loncar (2006), depression will be one of the top three leading causes of death in the world by the year 2030 (Mathers & Loncar, 2006). One of the most difficult periods in a woman’s life is the Postpartum period. Postpartum depression (PPD) is one of the common mental health states that affect women. A meta-analysis, including 59 studies from North America, Europe, Australia, and Japan, estimated the prevalence of postpartum depression as 13%. (Alasoom & Koura, 2014) While it is generally agreed that this illness can turn into major depression and carries a substantial risk of morbidity and death, it is an under-diagnosed and underrated illness. The impact of this has great consequences on the mother-child and the health care system and community.

Postpartum depression (PPD) is defined as a non-psychotic depressive episode that begins in or extends into the postpartum period. Symptoms include anxiety, guilt, negative maternal attitudes, and poor parenting self-efficacy. Arab women showed high rates of postpartum depression, with a prevalence between 16% and 35%. (Ayoub et al., 2020) In the United Arab Emirates, it was reported to be between 10% and 35% (Alhammadi et al., 2017; Ayoub et al., 2020; Green et al., 2006; Hamdan & Tamim, 2011). The United Arab Emirates has strong mother and child services. Nevertheless, there is no implemented screening and management program for mental health perinatally.

Early interventions targeting high-risk groups are found to be effective and can impact the lives of women, their children, and their families. Counseling, for example, is a safe and effective intervention to prevent perinatal depression, as per the Evidence Report and Systematic Review for the US Preventive Services Task Force. (O’Connor et al., 2019) As well, pharmacological agents are relatively safe, although the antidepressant medication, SSRI exposure was associated with increased risk later in children, this risk may be driven at least partly by the underlying severity of maternal illness or other confounding factors, more harm is anticipated as a consequence of underdiagnosis or undertreatment of PPD (Segre et al., 2023).

Multiple social determinants of health were among the key risk factors associated with the development of PPD which resulted in different epidemiology in different cultures (Alhasanat & Fry-McComish, 2015; Ayoub et al., 2020; Chi et al., 2016; Hamdan & Tamim, 2011; Lanes et al., 2011). This necessitates more research in identifying high-risk patients to enable early detection and management.

This study assesses the prevalence of the risk of postpartum depression among women in the Emirates of Abu Dhabi using a validated screening tool. As well as identifying the determinants of the risk of postpartum depression in this population. It is conducted in the well-child clinics where there is strong evidence to support the feasibility of active screening programs for parental postnatal depression as an integral part of postpartum care. (Segre et al., 2023)

## Materials and methods

### Study Design

A quantitative questionnaire-based cross-sectional study.

### Setting

It was conducted in the primary health care centers in the Emirates of Abu Dhabi.

### Study Population

Postpartum mothers who attended the well-established well-child clinics in the Emirates of Abu Dhabi accompanied their babies for well-child clinic visits at two months, four months, and six months. Mothers who are non-Arabic non-English speakers have been excluded.

A total of 240 women in the period of July 2017 until March 2018 completed the questionnaire after removing the uncompleted questionnaires, which were 15.

### Sample Size

The sample size was calculated to be 230 participants, with a confidence interval of 5.1% and a confidence level of 95%.

### The Questionnaire

The data collection form was a well-validated questionnaire to screen for postpartum depression, Edinburgh Postnatal Depression Scale EPDS (Bergink et al., 2011; Cox et al., 1987). The questionnaire used consisted of socio-demographic characteristics, and important histories such as obstetric, medical, and social histories. The main part of the questionnaire was the Edinburgh Post Partum Scale EPDS, which is the tool used to assess postpartum depression (Cox et al., 1987; Levis et al., 2020). The EPDS is a widely used, reliable, and validated screening tool for Postpartum Depression (PPD). It was specifically developed for screening at the primary health care level. The instrument has been translated and validated into 57 languages, including Arabic, and validated in 1997 in the United Arab Emirates. The EPDS screens for PPD using ten inventory questions investigating feelings occurring with the new mother within the previous seven days. Each question has four possible answers rated from 0 to 3. A test is “positive” for postpartum depression if the woman scores ten or more out of 30.15,16.

The EPDS was found in a recent systematic review and meta-analysis to have a sensitivity and specificity were 0.85 (95% confidence interval 0.79 to 0.90) and 0.84 (0.79 to 0.88) for a cut-off value of 10 or higher, 0.81 (0.75 to 0.87) and 0.88 (0.85 to 0.91) for a cut-off value of 11 or higher, and 0.66 (0.58 to 0.74) and 0.95 (0.92 to 0.96) for a cut-off value of 13 or higher, respectively. Accuracy was similar across reference standards and subgroups, including for pregnant and postpartum women (Levis et al., 2020).

Based on the score from EPDS, the resulting score was divided therefore into four categories. Category one has no risk for postpartum depression as their score was less than 10. Category two are those with mild risk for postpartum depression, as their score was from 10 to 12. Category three is the moderate risk for postpartum depression, which got a score of 13 to 15. Those who scored more than 15 were accounted as high risk for postpartum depression, and this is category four.

Therefore, scores less than ten were considered negative for postpartum depression, scores between 10 and 12 were considered low risk for postpartum depression, scores between 13 and 14 were considered moderate risk for postpartum depression, and scores of 15 or more were considered high risk of postpartum depression.

The tool was originally designed to be self-administered, but studies have shown that the directed interview EPDS and self-completed EPDS are equivalent screening techniques for postpartum depression. ^5^ The questionnaire is planned to be administered to the postpartum mothers by self, interview, nurse, or other qualified staff.

Women were recruited at any point when they brought their infants for a wellness check at 2, 4, or 6 months. The questionnaires were available at the well-child clinics distributed by the nurse and collected after being completed. For any questions, the nurse was available for guidance. Family medicine residents and practice improvement facilitators supported the nurses in the well-child clinics by training on the tool initially and then for any questions during the conduction of the study.

### Ethical Approval and Consent to Participate

The study was approved by the Ambulatory Healthcare Services IRB, which approved the study publication. All methods were carried out under relevant guidelines and regulations. no identifiers were collected from the subjects, and consent to participate was required to complete the survey.

### Consent Statement in the Ethics Approval and Consent to Participate

#### Informed Consent

was completed by all participants as per IRB guidelines and policy, and subjects were anonymized during collection and analysis.

### Statistical Analysis

Statistical analysis was performed using IBM SPSS statistics 19. Logistic regression was performed to assess the association of postpartum depression with different factors. P value less than 0.05 has been selected as a level of significance.

## Results

The study was more representative of non-UAE nationals, with 165 women being non-UAE nationals (67.9%) and 78 UAE national women (32.1). Table 1 shows the distribution of surveyed characteristics in relation to nationality (UAE and non-UAE nationals). Around 90% of the women were between 20 and 39 years old, with half of the women in the age group from 30 to 39. Regarding the educational level, 66.7% of the women had university degrees, and 35.8% of all women were employed. Two-thirds had two to five children, and most of them were living with their husbands and children, 83.9%.

**Fig. 1:**
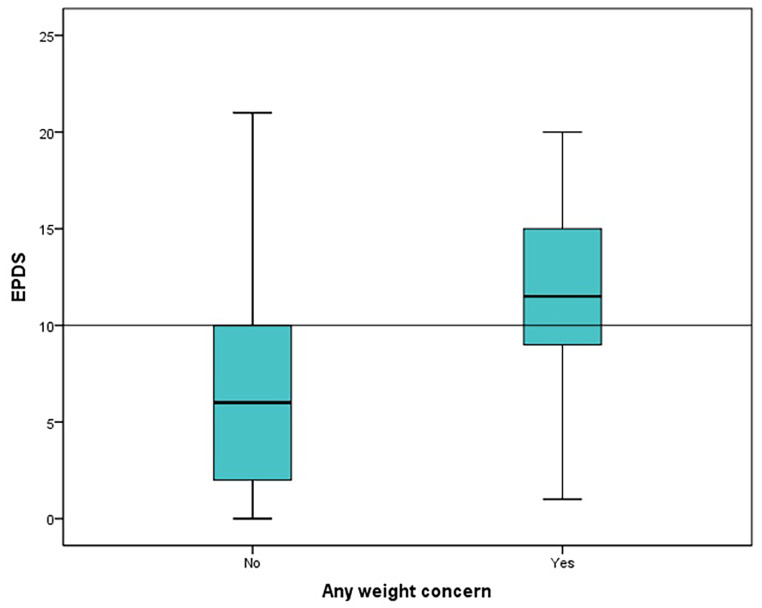
The relation between high EPDS score and the presence of any weight concerns

**Fig. 2:**
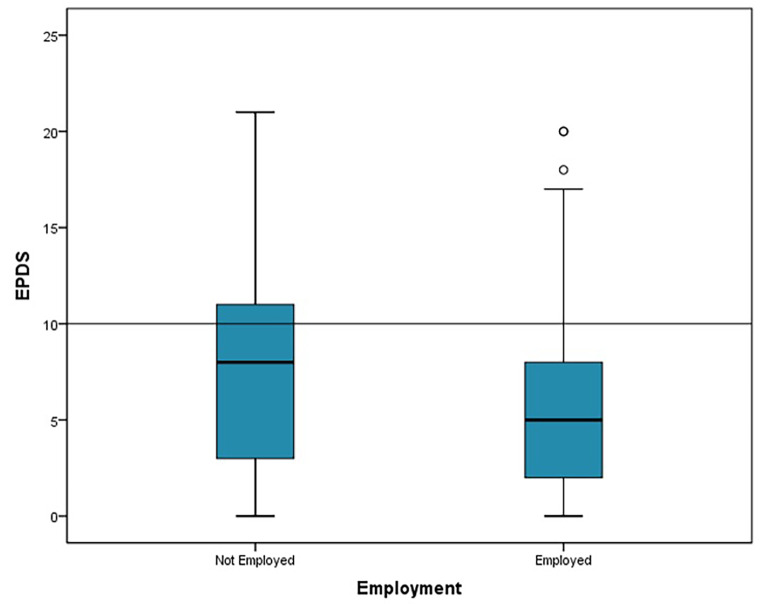
The relation between EPDS score and employment

One-third of the women visiting the well child care clinics in the Emirate of Abu Dhabi during the study period reported being at risk of postpartum depression; 10% had high risk, 7% had moderate risk, and 18% had mild risk. Using logistic and linear regression, Table 2, there was an association identified between postpartum depression risk using the Edinburg score and the presence of weight concern in the women surveyed and employment status OR 5.499(2.618–11.548) and OR 0.483 (0.246–0.951) respectively (*P* < 0.005) Figs. 1, 2. Women who have weight concerns are more likely to be at risk of PPD, and employed women are at lower risk than housewives.

**Fig. 3:**
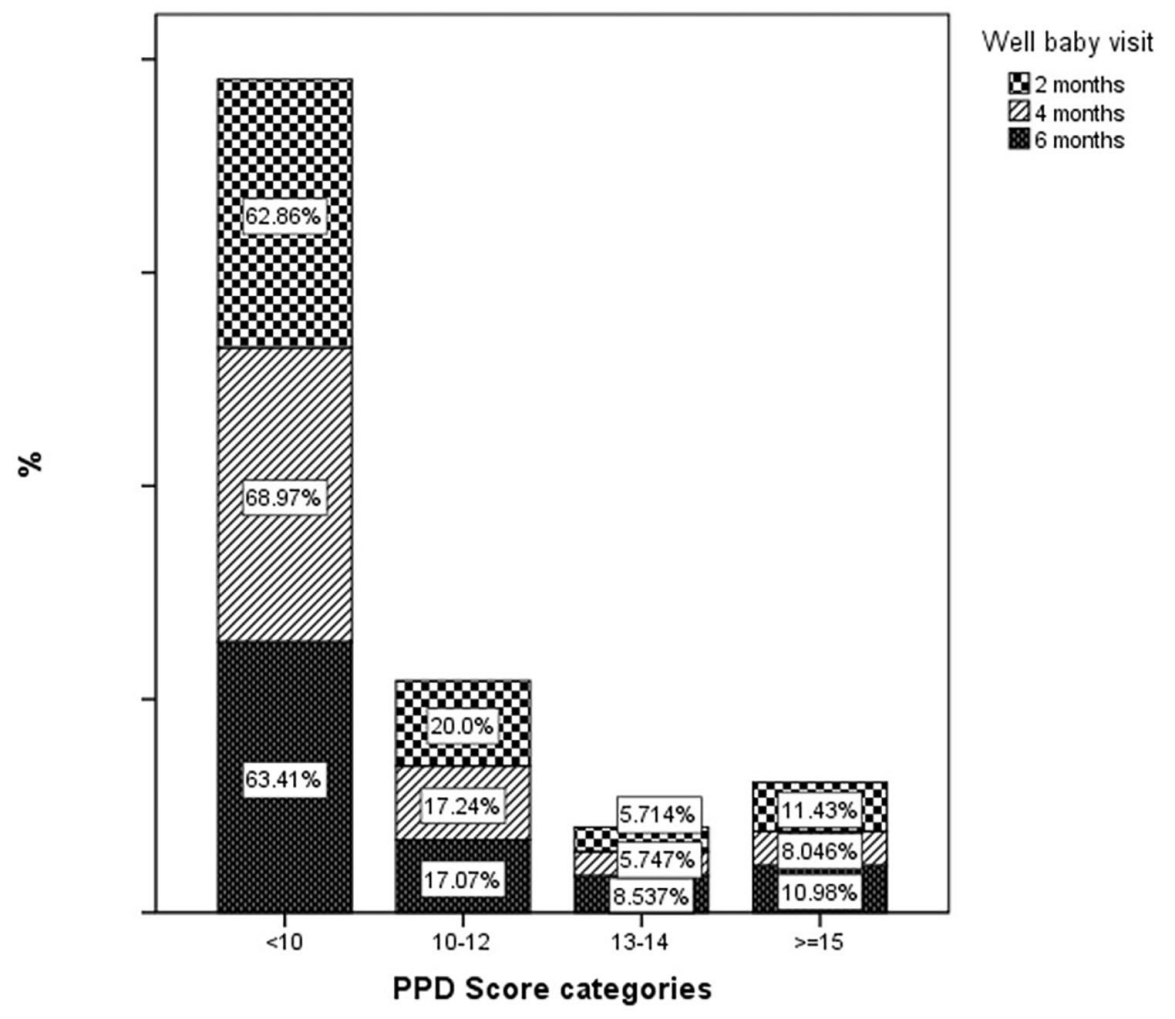
Postpartum Depression risk according to the well-child visits

**Fig. 4:**
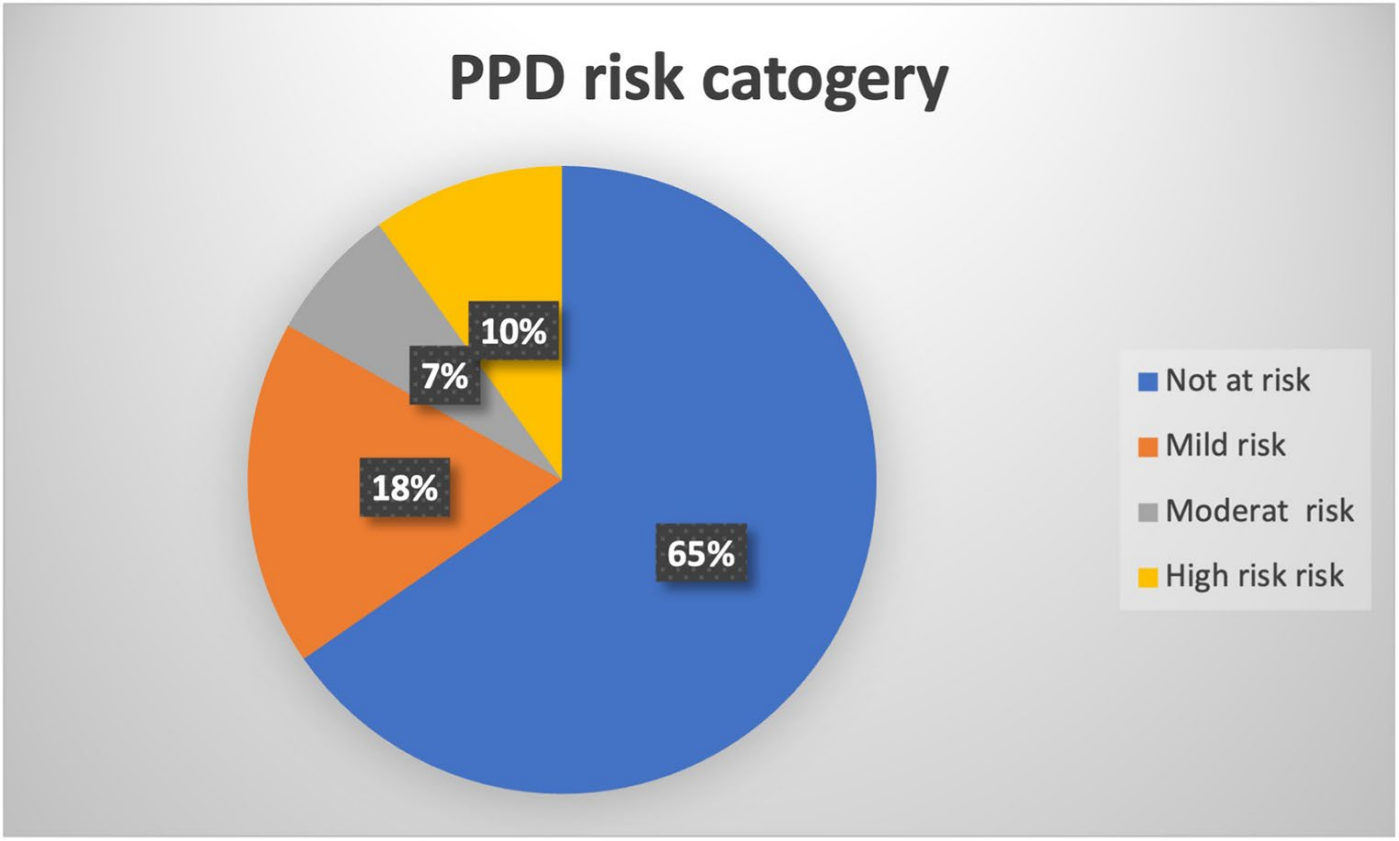
Prevalence of the risk of Postpartum Depression according to severity

Using logistic regression, nationality was not found to be a determinant factor of PPD. Figure 3 and 4. Additionally, other important factors that did not appear to be associated with the risk of PPD were living alone or with a family, infant feeding, gender of the infant, the mode of delivery, whether the pregnancy was planned or not, family income, number of children, educational Level of the women, and the time of the screening after delivery of the infant, 2, 4 or 6 months, Fig. 3.

Figure 4 shows the distribution of the study subjects by EPDS’s four risk categories. In category one, 158 (65%) were in this no-risk category. UAE national women *n* = 60 (78%) and non-UAE women *n* = 98(60%) scored less than 10. Thirty-nine of non-UAE (24%) and 4 of UAE national women (5%) scored in mild risk for PPD. In the moderate risk group, around 13(8%) and 3(6%) nonlocal and local women respectively scored between 13 and 15. In the high-risk group, around 14 (9%) of the non-UAE and 10 (13%) of the AUE scored more than 15.

Regarding self-harm, 9.1% of surveyed women have shown an indication of risk of self-harm. This is concluded from responses to question 10 (The thought of harming myself has occurred to me) in the EPDS; 0.4% responded with quite often, 3.3% sometimes, 5.4% hardly ever, and 90.9% Never (Table 3).

**Table 1:** Distribution of important population characteristics

Characteristic	Non-UAE	%	UAE	%
**Age**				
< 20	9	5.5	1	1.3
20–29	63	38.2	33	42.3
30–39	82	49.7	37	47.4
>=40	9	5.5	6	7.7
**Educational Level**				
Primary or less	8	4.8	0	0.0
Secondary	31	18.8	40	51.3
University and above	125	75.8	37	47.4
**Employment**				
Non employment	106	64.2	47	60.3
Employment	58	35.2	29	37.2
**Number of children**				
1	49	29.7	8	10.3
2 to 5	114	69.1	56	71.8
more than 5	1	0.6	13	16.7
**Family income**				
Not sufficient	13	7.9	3	3.8
Sufficient	120	72.7	57	73.1
Sufficient and saving	31	18.8	16	20.5
**Planned pregnancy**				
unplanned	71	43.0	44	56.4
planned	93	56.4	32	41.0
**Mode of delivery**				
Normal/ assisted vaginal delivery	92	55.8	56	71.8
Caesarean section	72	43.6	20	25.6
**Gender of baby**				
Female	68	41.2	42	53.8
Male	96	58.2	35	44.9
**Feeding of the baby**				
Exclusive breast feeding	75	45.5	23	29.5
Mixed	57	34.5	43	55.1
Formula milk	32	19.4	11	14.1
**Living with**				
Alone	1	0.6	1	1.3
Husband and children alone	145	87.9	59	75.6
Husband family	4	2.4	2	2.6
My family	14	8.5	15	19.2
Total	165	100.0	78	100.0

**Table 2:** Determinants of high edinburgh postnatal depression scale score

	B	Sig.	OR	95% C.I.	
Employment	-0.302	-0.147	0.015	-0.543	-0.060
Any weight concern	0.982	0.364	0.000	0.664	1.300

**Table 3:** Responses of participants to question 10 “The thought of harming myself has occurred to me”

		Number	%		
Never		219	90.9		
Hardly ever		13	5.4		
Sometimes		8	3.3		
Yes, quite often		1	0.4		
Total		241	100.0		

## Discussion

This study revealed a PPD risk prevalence of (35%). One-third of the sample of the women in the Emirates of Abu Dhabi surveyed had a risk of postpartum depression, with 3.7% responding quite often or sometimes to the intention to harm themselves. Additionally, one in ten of the surveyed women were in the higher risk category (scored more than 15). A previous study done in the United Arab Emirates in 2006, which recruited Emirati women in a government maternity hospital in Abu Dhabi (Green et al., 2006) found that at the 3-month postpartum period, the prevalence of postpartum depression was 22%, with another 22% falling in the Borderline Depression category, and at the 6-month postpartum period, the prevalence of postpartum depression was 12.5%, where another 19.6% had Borderline Depression category. In two studies done in the same interval as this study, both found a similar prevalence range of 33% and 35% risk of PPD (Hanach et al., 2023) (Alhammadi et al., 2017). On the other hand, in an earlier study on PPD prevalence from Sharjah in 2011, the prevalence was reported to be only 10% by Hamdan and Tamim (Hamdan & Tamim, 2011), which may indicate an increasing risk.

A large meta-analysis done in 2018, estimating the global and national prevalence of postpartum depression with the identification of economic, health, social, or policy factors associated with national postpartum depression prevalence, showed that the global pooled prevalence of postpartum depression was 17.7%, indicating UAE reported prevalence to be high compared to other countries. A similar trend is seen in Saudi Arabia, where in 2014, a study from Saudi Arabia reported a prevalence of 17.8% in Dammam, and another study in the capital, Riyadh, found a prevalence of 38.50%, which may reflect a regional difference. (Alasoom & Koura, 2014) (Al Nasr et al., 2020). In Australia, the prevalence was less, 5.5% of women screened had a postnatal EPDS > 9 while a recent systematic review reported prevalence in the Arab world between 15 and 25% (Ayoub et al., 2020).

Regarding determinants of postpartum depression, the only significant association identified in this study was between postpartum depression risk using the Edinburgh Postnatal Depression Scale EPDS and the presence of weight concern in the women surveyed and the employment status. In Hanach study Husband’s employment, husband’s support, and living in own house were associated with a lower risk of postpartum depression. Maternity leave of more than 3 months increased the risk of depression during the first 3 months postpartum but these were not significant in the same study (Hanach et al., 2023). Some forms of social isolation may be minimized by being employed or the fact that working women are involved in activities that support their well-being and mental health. More qualitative inquiry is required to find factors that may help in PPD prevention and management. In a Meta-synthesis of qualitative studies, Loneliness appears to play a central role in the experience of perinatal depression based on the frequency with which it emerged in women’s accounts. (Adlington et al., 2023)

Women who have weight concerns are more likely to be at risk of postpartum depression compared to women without weight concerns. This is confirmed in a meta-analysis of observational studies which found that excessive and inadequate gestational weight gain was significantly associated with a higher risk of developing PPD. Suggesting that strengthening the prevention and intervention of excessive and inadequate weight gain during pregnancy will promote maternal and infant health better outcomes (Qiu et al., 2022). Comparing these findings with the findings from the previous study done in the United Arab Emirates in 2006, they reported a similar relation. The risk of postpartum depression was higher for those with poor self-body image with a view of weight.

On the other hand, they found that other factors are associated with postpartum depression, including not breastfeeding, giving birth to the first child, having a poor relationship with the mother-in-law, and older age at marriage, which were not found to be significantly influential in this study (Green et al., 2006). This study found as well that there was no association identified between postpartum depression and age, educational level, family income, planned pregnancy, mode of delivery, gender of infant, breastfeeding, and the people who live with the mother.

Nevertheless, other studies have reported an association with other variables. For example, a Study done in Canada in 2011 among 6,421 Canadian women using the Edinburgh Postnatal Depression Scale showed that a mother’s stress level during pregnancy, the availability of support after pregnancy, and a prior diagnosis of depression were the strongest significant association with the development of postpartum depression. (Lanes et al., 2011) Another study done in China in 2016, where A total of 506 mothers 23 years of age and older who were within three years postpartum completed the online survey, showed that there are factors contributing to the development of postpartum depression including; education level, family income, preparation for pregnancy, a history of depression, amount of time spent with their husbands, relationships with husbands, parents, and parents-in-law, and a more anxious attachment style were strongly related to a higher risk of postpartum depression. (Chi et al., 2016)

From this data, a screening program for postpartum depression in primary health care centers among mothers coming already for their well child checkup, for example, is a necessity. Through this program, we will have the means to address maternal mental health. As well we will spot the light on a hidden area in women’s health, which also affects the infant’s well-being in the long run.

Lessons learned from this study is that we need to strengthen mental health services in Ambulatory Health Service (AHS) by increasing the availability of psychologists, psychiatrists, and social workers side by side working with family physicians in order to provide better care from all aspects that the patient may need.

A systematic review done in 2016 studying the evidence on the effectiveness of screening for postpartum depression in well-child clinic setting compared to no screening, regarding mother and child outcomes showed that 4 out of 6 studies demonstrated an increase in the detection rate of depressive symptoms, referral and treatment, and thus supporting the potential of screening in well infant clinic setting with positive evidence. (van der Zee-van den Berg et al., 2017) Through a structured care system through frequent contact with new mothers up to 1 year of postpartum by regular appointment and, if needed, by phone calls to be sure that we are not missing any chance to help these mothers. We will be able to detect and respond to these women Major complications on women, babies, and families will be prevented.

More research should be conducted on the area of postpartum care in general, not only to look at mental health but also to address any medical problems and to provide data about the implications of postpartum care programs in primary health care centers.

A limitation of this study is the lack of confirmation of the diagnosis as this was an anonymous study. Nevertheless, a strength of this study is the inclusion of all Abu Dhabi centers, and that these centers provide free well-child healthcare services and therefore there is no restriction to any population groups. As well, the UAE context with a highly accessible healthcare system and a high number of extended families is likely to be favorable for future studies to build on it to assess other barriers and facilitators of early diagnosis and care-seeking behavior.

## Conclusion

EPDS is recommended to be used routinely to screen women in the postnatal period. This high prevalence of risk of postpartum depression in the UAE (One in three women) calls for a well-prepared healthcare system and community. Healthcare providers need to be prepared with better knowledge, practice, and management strategies to care for these women, for early identification and management. Further studies should be undertaken to achieve effective strategies to reduce the incidence of this condition.

## Data Availability

Data is available on request sent to the corresponding author.
